# Ultrafast manipulation of topologically enhanced surface transport driven by mid-infrared and terahertz pulses in Bi_2_Se_3_

**DOI:** 10.1038/s41467-019-08559-6

**Published:** 2019-02-05

**Authors:** L. Luo, X. Yang, X. Liu, Z. Liu, C. Vaswani, D. Cheng, M. Mootz, X. Zhao, Y. Yao, C.-Z. Wang, K.-M. Ho, I. E. Perakis, M. Dobrowolska, J. K. Furdyna, J. Wang

**Affiliations:** 1 Department of Physics and Astronomy and Ames Laboratory-U.S. DOE, Iowa State University, Ames, Iowa 50011 USA; 2 Department of Physics, University of Notre Dame, Notre Dame, IN 46556 USA; 3 Department of Physics, University of Alabama at Birmingham, Birmingham, AL 35294-1170 USA

## Abstract

Topology-protected surface transport of ultimate thinness in three-dimensional topological insulators (TIs) is breaking new ground in quantum science and technology. Yet a challenge remains on how to disentangle and selectively control surface helical spin transport from the bulk contribution. Here we use the mid-infrared and terahertz (THz) photoexcitation of exclusive intraband transitions to enable ultrafast manipulation of surface THz conductivity in Bi_2_Se_3_. The unique, transient electronic state is characterized by frequency-dependent carrier relaxations that directly distinguish the faster surface channel than the bulk with no complication from interband excitations or need for reduced bulk doping. We determine the topological enhancement ratio between bulk and surface scattering rates, i.e., *γ*_BS_/*γ*_SS_ ~3.80 in equilibrium. The ultra-broadband, wavelength-selective pumping may be applied to emerging topological semimetals for separation and control of the protected transport connected with the Weyl nodes from other bulk bands.

## Abstract (web summary)

It remains challenging on how to selectively control terahertz conductivity at surface from the bulk contribution in topological insulators. Here, Luo et al. discover and manipulate topologically enhanced surface transport due to helical spin structure using mid-infrared and terahertz ultrafast photoexcitations.

## Introduction

The recent discovery of topology-protected charge transport of three-dimensional (3D) TIs has led to a promising platform for exploring both fundamental topological quantum phenomena and technological applications^1–7^. The emergent behaviors associated with this exotic state of matter, such as spin-locking, helical spin structure, topological invariant, chiral anomaly, and dissipationless currents^8–13^, offer new perspectives for achieving transformational technological applications in spintronics and quantum sensing, computing, and communications^3^ beyond the current technological limit. One of the current frontiers for topological phenomena lies in the fundamental challenge of how to disentangle and manipulate symmetry-protected transport from bulk conduction. On one hand, development and optimization of hot electron transistors and modulators operating at ultra-high THz frequencies will significantly benefit from the direct probing of ultrafast THz charge transport on the surface and from a better understanding of the fundamental effects of topological spin-locking on carrier scattering. Although the existence of a helical Dirac spectrum has been well established in electrical transport^14^ and photoemission^8,15,16^, ultrafast control of THz helical spin transport is much less known. This is due, in part, to the limitations of conventional transport characterization methods that are incapable of probing frequency-dependent conductivity characterized by femtosecond (fs) in time and THz in energy scales, especially with low frequency photon pumping^17–19^. On the other hand, in 3D TIs, the coexistence and mutual scattering of Dirac and bulk carriers lead to an intertwined response. Despite recent observations from static THz measurements of TI samples with significantly reduced doping and defects^20,21^, it is still challenging to selectively control the intrinsic surface dynamics from the bulk contribution for samples that exhibit unintentional doping into bulk states.

The transport properties of 3D TIs are determined by carriers in narrowly gapped bulk bands and by gapless surface states protected by the time-reversal symmetry. Although the spin-momentum locking of Dirac electrons increases the conductivity from atomically-thin surface states, its contribution can still be masked by the high density carrier conduction in static transport measurements, at either bulk or interface states, even in films with tens of nm thickness. Consequently, very few experimental techniques are capable of isolating and studying the intrinsic topological transport on the surface, especially for high Fermi energies. Sophisticated characterization and sample synthesis are involved, e.g., anisotropic magneto-transport by adjusting magnetic field directions^14^ and significantly reduced doping and thickness by thin film engineering^20–29^. There has been considerable progress yet with complications, e.g., limited applicability or difficulty in the interpretation. For example, intrinsic Dirac-cones could be contaminated with possible gap opening in very thin samples. The separation of surface and bulk/interface contributions to the static THz conductivity spectra has to rely on reduced doping.

Recently, ultrafast THz conductivity has been shown to be promising by providing time resolution able to distinguish the surface from the bulk responses after suddenly driving the system out-of-equilibrium^28,30^. However, the high photon frequency pumping at 1.55 eV used so far introduces interband excitations from valence to conduction and other high lying bands at both surface and bulk. Such coupling causes population transfer between surface and bulk bands and enhanced scattering between high-energy states. Selective mid-IR and THz pump-THz probe spectroscopy used in this work represent powerful and versatile tools that allow us to measure and control surface transport in TIs, even for Fermi level *E*_F_ into the bulk bands.

**Fig. 1:**
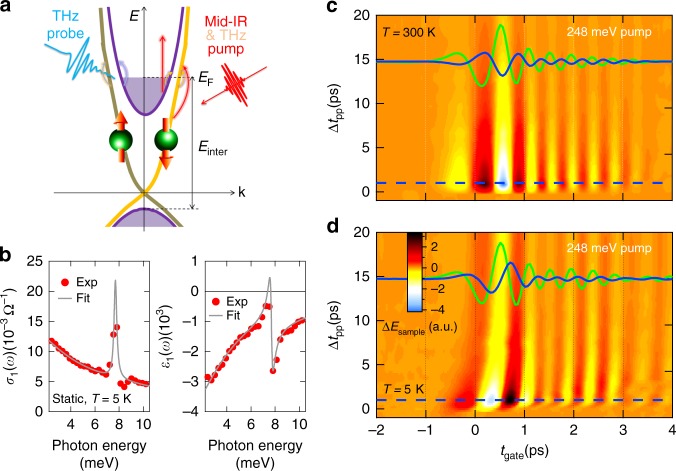
Ultrafast mid-IR/THz pump and THz probe spectroscopy for Bi_2_Se_3_. **a** Schematic of the surface and bulk electronic band structures of the Bi_2_Se_3_ film with Fermi level (*E*_F_) indicated, illustrating mid-IR/THz intraband excitations and THz conductivity detection. **b** Static THz spectra of *σ*_1_(*ω*) and *ε*_1_(*ω*) (red dots) of the Bi_2_Se_3_ film at *T* = 5 K. Shown together are the theoretical fitting (gray lines) using the composite THz model from Eq. (1). **c**, **d** A 2D false-color plot of pump-induced THz field changes Δ*E*_sample_ as a function of gate time *t*_gate_ (horizontal axis) and pump-probe delay Δ*t*_pp_ (vertical axis) after 248 meV (5 μm) photoexcitation of the sample at **c** *T* = 300 K and **d** *T* = 5 K, respectively. Plotted together are the corresponding static THz fields *E*_sample_(*t*_gate_) (green curves) and their pump-induced changes Δ*E*_sample_(*t*_gate_) at Δ*t*_pp_ = 1 ps (blue curves, ×5, from the cut positions as marked by the blue-dashed lines), in order to compare their relative amplitude changes and phase shifts at different temperatures

We emphasize several strategic advantages of low frequency pumping scheme like ours, as illustrated in Fig. 1a. First, in contrast to interband photoexcitation that couples high-energy electronic states between two bands, THz and mid-IR photoexcitation mainly excites intraband transitions near *E*_F_. Here the photon energy is below the insulating bandgap and interband transition energy, which suppresses many other charge transfer channels. This allows measurement of intrinsic scattering rates and strongly constrains the fitting parameters to scattering rates only, as discussed later. Additionally, our time resolution can separate intrinsic surface from bulk conductivity via their different hot carrier cooling times, i.e., resolve the issue directly in the time-domain. The extracted spectra fully characterize THz response functions as the complex frequency-dependent conductivity. In this way we can determine the intrinsic surface electron scattering rates and their ultrafast dynamics, complimentary to commonly used transport^14^, and photoemission^8,16^ measurements. Finally, comparison of the conductivity for wavelength-selective photoexcitations below and above the interband transition enabled by tuning the pump photon energy allows the optical control of carrier dynamics of surface and bulk bands in a selective way. However, in contrast to the several interband photoexcitation studies performed in TIs^28,30^, mid-IR, and THz pump-induced conductivity experiments have not been carried out so far in TIs.

Here we report ultrafast THz charge transport and dynamics in n-type Bi_2_Se_3_ that arise from nonequilibrium Dirac surface and bulk transient states induced by intraband mid-IR and THz photoexcitation. We observe frequency-dependent carrier cooling times of photoinduced THz conductivity that clearly differentiate surface from bulk contributions and allow determination of their scattering rates. We show that the topological enhancement of surface transport suppresses the surface electron scattering rate as compared to the bulk, i.e., *γ*_BS_/*γ*_SS_~3.80 in equilibrium. This result is consistent with surface helical spin transport in the presence of short-range disorder^31^. Moreover, the clear similarities between mid-IR and THz pumping and their distinct difference from high-photon-energy cases clearly show: (1) ultrafast manipulation of the surface and bulk THz conductivities via wavelength-selective pumping; (2) intraband vs. interband excitation mechanisms driven by low and high-photon-energy pumping. These are distinctly different from any known THz transport measurements so far, which have used either high-energy optical frequency photoexcitation or only detected time-averaged properties. The intraband, mid-IR/THz-induced THz conductivity provides a powerful method for isolating the surface transport channel in TIs—unhindered by complications from interband pumping or high density doping into bulk states. The distinct spectral-temporal characteristics obtained in this way in TIs may be extended to study and understand much broader topological phenomena^32^.

## Results

### Frequency-dependent dynamics of THz conductivity spectra

The Bi_2_Se_3_ thin film sample, 50 nm thick, is grown by molecular beam epitaxy on a 0.5 mm thick sapphire substrate. The static THz conductivity spectra are shown in Fig. 1b. The details about THz data analysis, sample preparation, and experimental setup are described in the Methods. We characterize the nonequilibrium THz responses of the Bi_2_Se_3_ thin film by extracting the real parts of the transient conductivity *σ*_1_(*ω*, Δ*t*_pp_) and dielectric function *ε*_1_(*ω*, Δ*t*_pp_) as a function of both the frequency *ω* and the pump-probe delay time Δ*t*_pp_. These spectra describe the dissipative and inductive parts of the response functions of quasi-particles, respectively, and are extracted from raw THz fields in time-domain.

**Fig. 2:**
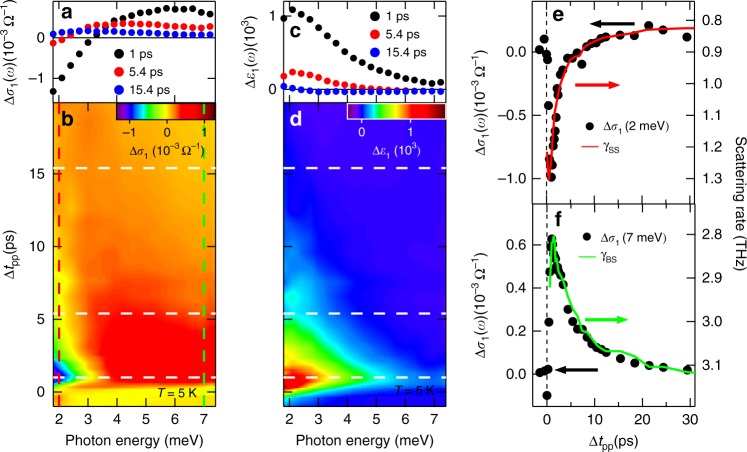
Frequency-dependent dynamics of THz spectra at low temperature. Pump-induced THz spectra of **a**, **b** Δ*σ*_1_(*ω*) and **c**, **d** Δ*ε*_1_(*ω*) after 248 meV (5 μm) photoexcitation with fluence 12 μJ cm^−2^ at *T* = 5 K as a function of pump-probe delay Δ*t*_pp_. **a** and **c** show the THz spectra from three cut positions from the corresponding 2D plots of **b** and **d**, respectively, at Δ*t*_pp_ = 1, 5.4, and 15.4 ps, as indicated by the white-dashed lines. **e** THz conductivity at 2 meV, i.e., Δ*σ*_1_(2 meV) (black dots, left axis), as a function of Δ*t*_pp_, from the frequency-cut position in **b** as indicated by the red-dashed line. The scattering rate of the surface state *γ*_SS_ (red curve, right axis) obtained from theoretical fitting using Eq. 1 is plotted together to compare relaxation dynamics. **f** Similar to **e**, THz conductivity at 7 meV, Δ*σ*_1_(7 meV) (black dots, left axis) and the scattering rate of bulk state *γ*_BS_ (green curve, right axis) are plotted. Δ*σ*_1_(7 meV) is from the frequency-cut position in **b** as indicated by the green-dashed line. As shown clearly, the relaxation dynamics of the surface (bulk) scattering rate matches with that of THz conductivity at 2 meV (7 meV) very well. This frequency-dependent THz relaxation dynamics indicates surface (bulk) state is more sensitive to low (high) THz frequency

The pump-induced raw THz fields in Fig. 1c, d exhibit a distinctly different temperature dependence. At 300 K, the transmitted field change Δ*E*_sample_ has a *π* phase shift relative to the static field *E*_sample_ without any other frequency- and time-dependent changes. This behavior is characteristic of an absorption-dominated response, i.e., a large increase in *σ*_1_(*ω*). On the other hand, at low temperature *T* = 5 K, a large inductive response *ε*_1_(*ω*) appears in addition to the dissipative one, as manifested by a significant frequency- and time-dependent THz field reshaping besides the dominant *π* phase shift as in the 300 K case. These distinct spectral-temporal characteristics give rise to several salient features in the extracted THz response functions Δ*σ*_1_(*ω*) and Δ*ε*_1_(*ω*), shown in Fig. 2a–d, that allow us to directly separate the intrinsic surface and bulk transport contributions to the conductivity from the experimental data without fitting.

The first feature, shown in Fig. 2a, b, is that the pump-induced THz conductivity spectra Δ*σ*_1_(*ω*) extracted from the measured time-dependent THz fields exhibit a clear bipolar behavior with frequency-dependent cooling times under intraband excitation (248 meV) at low temperature (5 K). Specifically, as shown by comparing three time-cut spectra (white-dashed lines in Fig. 2b), Δ*σ*_1_(*ω*) at early times (Δ*t*_pp_<5 ps) is characterized by a strong bleaching, i.e., a negative conductivity change, in the low frequency 2–3 meV range. This changes to a strong absorption, i.e., positive conductivity change, towards high frequencies up to ~7 meV, as seen in the 1 ps trace (black dots in Fig. 2a). At Δ*t*_pp_>5ps, the low frequency bleaching has quickly diminished, and the transient spectra are dominated by the high frequency absorption with longer cooling times, e.g., as shown in the 5.4 ps (red dots) and 15.4 ps (blue dots) traces. The frequency cuts of the Δ*σ*_1_(*ω*) spectra from Fig. 2b are summarized in Fig. 2e (at *ω* = 2 meV) and 2f (at *ω* = 7 meV) (black dots) as a function of Δ*t*_pp_. Clearly, the cooling times depend on the probe frequency, i.e., the faster (slower) cooling for ultrafast THz conductivity change at low (high) frequency. The characteristic cooling time for the 2 meV probe is *τ*_SS_ = 1.48 ± 0.14 ps, significantly shorter than that for the 7 meV probe *τ*_BS_ = 5.30 ± 0.18 ps (the uncertainty is from single exponential fitting). Such a frequency-dependent decay of THz electron transport is consistent with the significant THz pulse reshaping seen in Fig. 1d, which indicates more than one cooling channel of hot electrons.

Second, to understand these experimental results, we compare them with prior ultrafast THz responses from photoexcited Bi_2_Se_3_ samples. A decrease in their thickness has been shown to induce a transition to a surface-like, pronounced bleaching behavior that differs from a bulk-like, absorption one^30^. The latter is expected from the ionized impurity scattering in the bulk, which decreases scattering rate after pumping at low temperatures^33^ and gives rise to a positive Δ*σ*_1_(*ω*) towards zero frequency (see below for details). Therefore our results show that THz transport and dynamics of surface and bulk fermions can be selectively separated in time and measured by tuning probe frequency. Most intriguingly, upon closer scrutiny, the time-dependent scattering rates of surface Dirac (red line) and bulk (green line) fermions in Fig. 2e, f match very well with the conductivity dynamics at 2 meV and 7 meV, respectively. These scattering rates are extracted by fitting the time-dependent conductivity data as discussed later. This clearly shows that the experimentally obtained Δ*σ*_1_(*ω*) probed at 2 meV can be used to directly measure surface transport dynamics, distinctly different from bulk conduction (at 7 meV), without reference to theoretical models. In between 2 and 7 meV, Δ*σ*_1_(*ω*) dynamics consist of mixed responses due to competing surface and bulk states as expected.

**Fig. 3:**
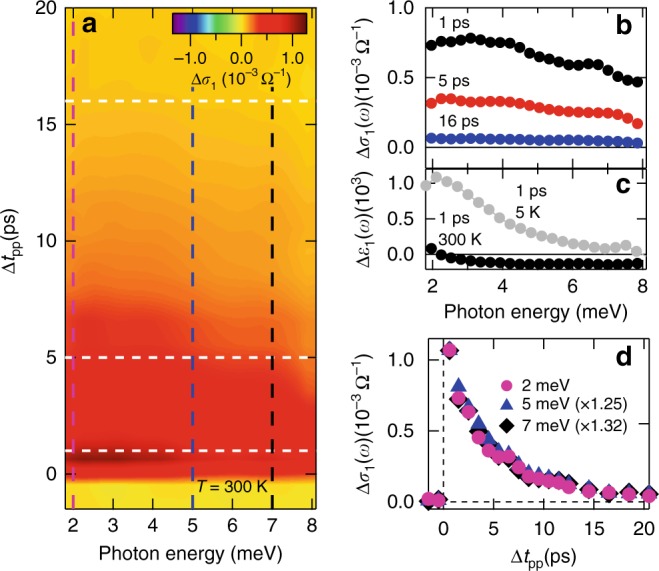
Frequency-independent dynamics of THz spectra at high temperature. **a** Pump-induced THz spectra of Δ*σ*_1_(*ω*) after 248 meV (5 μm) photoexcitation with fluence 12 μJ cm^−2^ at *T* = 300 K as a function of pump-probe delay Δ*t*_pp_. **b** THz conductivity Δ*σ*_1_(*ω*) from three cut positions from **a**, at Δ*t*_pp_ = 1, 5, 16 ps, as indicated by the white-dashed lines. **c** THz spectra of Δ*ε*_1_(*ω*) at Δ*t*_pp_ = 1 ps for 5 K (gray dots) and 300 K (black dots), the amplitude of which is much smaller at high temperature, opposite to low temperature ones in Fig. 2c, d. **d** The comparison of THz conductivity Δ*σ*_1_(*ω*) at three frequencies: 2, 5, and 7 meV, as a function of Δ*t*_pp_ (scaled to match amplitude), from three frequency-cut positions from **a** as indicated by magenta, blue, and black dots, respectively. The frequency-independent relaxation dynamics of Δ*σ*_1_(*ω*) indicates the strong coupling of surface and bulk states that make them decay together at high temperature

The third feature is that increasing the lattice temperature leads to an induced absorption behavior over the entire frequency range measured with a frequency-independent single cooling time, as shown in Fig. 3. Strikingly, this high-temperature behavior is opposite to the low-temperature conductivity responses in Fig. 2. This is consistent with the different, raw THz fields Δ*E*_sample_ between *T* = 5 K and 300 K seen in Fig. 1c, d. Specifically, the pump-induced Δ*σ*_1_(*ω*) spectra at 300 K, shown as 2D false-color plot in Fig. 3a, are all positive, Δ*σ*_1_(*ω*) > 0, without the negative bleaching component over the measured time and spectral range. Particularly, Δ*σ*_1_(*ω*) has similar amplitude at 5 K and 300 K as shown in Figs. 2a and 3b, but Δ*ε*_1_(*ω*) at 300 K is decreased to approximately one order of magnitude smaller than its counterpart at 5 K, as shown in Figs. 2c and 3c (see also the Supplementary Note 1). This indicates large photoinduced conductive response in comparison with inductive one at the elevated temperature. Since phonon-mediated coupling and charge transfer between surface and bulk are activated above the Debye temperature at 182 K^15^, the dominance of positive Δ*σ*_1_(*ω*) at 300 K is consistent with our conclusion that the bulk transport and surface-bulk charge transfer dominate at high temperatures and lead to the induced absorption. This suppresses the induced bleaching from the surface seen at 5 K in Fig. 2a. On the other hand, the frequency-dependent cooling times previously seen at 5 K are no longer present at the elevated temperature. As shown in Fig. 3d the THz conductivities Δ*σ*_1_(*ω*) at *ω* = 2, 5, and 7 meV as a function of pump-probe delay Δ*t*_pp_, now decay with the same time at 300 K. The cooling time of ~4.8 ps is similar to the bulk state cooling time *τ*_BS_ observed at 5 K. These observations corroborate again our claim that the low frequency conductivity at 2 meV, with faster cooling time observed at 5 K, comes from surface transport that is separated from the bulk channel. Furthermore, these distinct differences between high and low temperatures in ultrafast charge transport suggest that surface-bulk charge transfer in TIs is suppressed at low temperature.

### THz spectra analysis and fitting

To put these physical pictures on a strong footing, we quantitatively simulate the experimentally determined THz response functions Δ*σ*_1_(*ω*) and Δ*ε*_1_(*ω*). By extracting the temporal evolution of surface and bulk conductivity spectra, we obtain the intraband scattering rates and ultrafast dynamics of both Dirac (red line) and bulk (green line) fermions, as presented in Fig. 2e, f, respectively. A theoretical model consisting of contributions with Drude and Lorentzian lineshapes is used to reproduce the measured THz response functions. Specifically, two Drude terms account for surface state (SS) and bulk state (BS) plus one phonon oscillator centered at *ω*_0_ ~ 7.7 meV (1.9 THz),

1 $$\tilde \varepsilon (\omega ) =	 \mathop {\sum}\limits_{j = {\mathrm{SS,BS}}} \tilde \varepsilon _j^{{\mathrm{Drude}}}(\omega ) + \varepsilon _{{\mathrm{ph}}}(\omega ) = \varepsilon _\infty - \mathop {\sum}\limits_{j = {\mathrm{SS,BS}}} \frac{{(\omega _{\mathrm{p}})_j^2}}{{\omega ^2 + i\omega \gamma _j}}\\ 	+ \frac{F}{{(\omega _0)^2 - \omega ^2 - i\omega {\mathrm{\Gamma }}}},$$

where *ε*_∞_ is the background electrical permittivity and the plasma frequency $(\omega _{\mathrm{p}}^2)_j = ne^2/\varepsilon _0m^ \ast$ is proportional to the density of charge carriers of surface (*n*_SS_, *j* = SS) and bulk (*n*_BS_, *j* = BS) electrons. *ω* and *e* are the frequency and electron charge. *ε*_0_ and m^*^ are the vacuum electrical permittivity and carrier effective mass. *γ*_*j*_ is the scattering rate of surface (*j* = SS) and bulk (*j* = BS) carriers. In the last term of Eq. 1, *F* denotes the effective transition strength of the optical phonon with resonance frequency *ω*_0_ and scattering rate Γ. Such a composite THz response model provides an excellent agreement with the pump-induced transient THz spectra over the entire measured pump-probe delays and spectral range as shown in Fig. 4 (cyan lines). Such fit is achieved by only varying the scattering rates *γ* of surface and bulk carriers, i.e., the charge carrier densities of surface (*n*_SS_) and bulk (*n*_BS_) states remain constant in the fits. Please also note that the optical phonon resonance ~7.7 meV is fitted by *F*, *ω*_0_, and Γ of the Lorentzian oscillator, which only affects locally the feature near the resonance. Examples of typical fits are shown in Fig. 4 at various time delays, Δ*t*_pp_ = 1 ps, 9.4 ps, and 15.4 ps. The model fit (cyan lines) is divided into the sum of the surface (green-dashed lines), bulk (blue-dashed lines), and phonon (magenta-dashed lines) responses. Detailed fitting parameters are listed in the Supplementary Note 5.

**Fig. 4:**
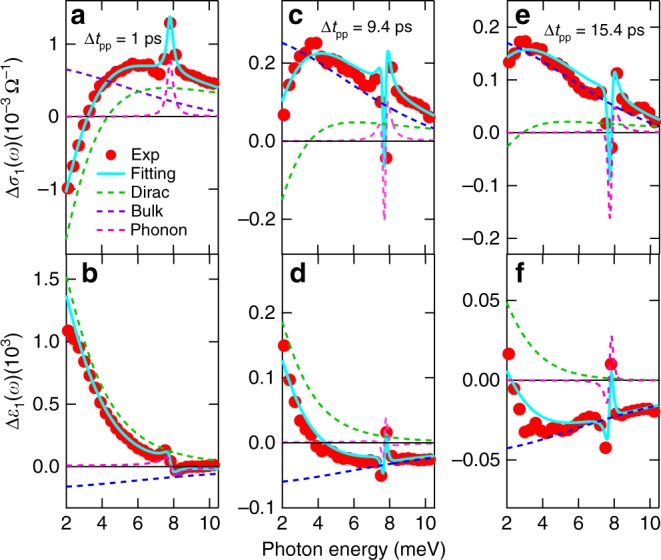
Theoretical fitting of the pump-induced THz spectra. **a**, **b** The fitting of Δ*σ*_1_(*ω*) and Δ*ε*_1_(*ω*) for 248 meV (5 μm) photoexcitation with fluence 12 μJ cm^−2^ at *T* = 5 K and pump-probe delay Δ*t*_pp_ = 1 ps. The experimental results (red dots) are fitted very well by the THz model from Eq. 1 (cyan lines), which consists of three individual components, i.e., the Dirac surface state (green-dashed lines), bulk state (blue-dashed lines), and optical phonon mode (pink-dashed lines). The fitting results for **c**, **d** Δ*t*_pp_ = 9.4 ps and **e**, **f** Δ*t*_pp_ = 15.4 ps are plotted in the same manner as **a**, **b**

**Fig. 5:**
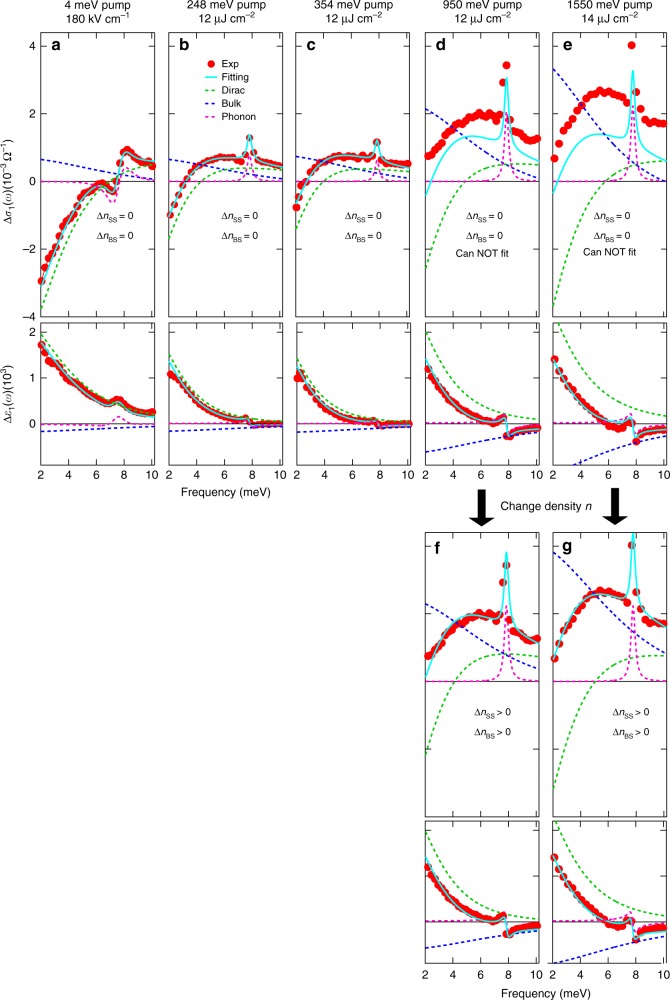
Ultrafast THz spectra by wavelength-selective pump from THz to visible. THz spectra for several pump photon energies: **a** 4 meV, **b** 248 meV, **c** 354 meV, **d**–**f** 950 meV, and **e**, **g** 1550 meV at Δ*t*_pp_ = 1 ps. Figures for Δ*σ*_1_ and Δ*ε*_1_ have the same *x* and *y* axis ranges, respectively, for fair comparison. The THz spectra for **a** 4 meV, **b** 248 meV, and **c** 354 meV pump can be fitted well by keeping the SS and BS carrier densities constant (Δ*n*_SS_ = 0 and Δ*n*_BS_ = 0) and only changing the scattering rates of SS and BS. However, the THz spectra cannot be fitted in the same way for **d** 950 meV and **e** 1550 meV pump. Instead, they have to be fitted by changing both the carrier densities (Δ*n*_SS_ > 0 and Δ*n*_BS_ > 0) and the scattering rates of SS and BS, as shown in **f** and **g**

We like to emphasize three key observations from the quantitative fitting. First, while the static THz spectra, shown in Fig. 1b, can be fitted equally well with either single or multiple Drude components, the transient THz spectra, shown in Fig. 4, can only be fitted by the two-Drude model. This is the case because the strong constraint imposed by the requirement to simultaneously describe both the conductivity Δ*σ*_1_(*ω*) and the dielectric function Δ*ε*_1_(*ω*) over broad temporal and spectral ranges, shown in Fig. 2a–d, instead of just fitting the lineshape as in the static case. Second, an even stronger constraint comes from the intraband, mid-IR/THz excitation scheme that most likely conserves the number of surface and bulk quasi-particles after the photoexcitation, i.e., carrier densities of both surface and bulk states are kept constant in the fitting, Δ*n*_SS_ = 0 and Δ*n*_BS_ = 0 (more details and discussions in Fig. 5). Remarkably, by simply varying the surface and bulk scattering rates we are able to consistently reproduce the experimental results, as shown in Fig. 4, and extract the scattering rates (or inverse transport lifetimes that should not be confused with hot carrier cooling times) *γ*_SS_ (red line), *γ*_BS_ (green line), and their cooling dynamics in Fig. 2e, f. On the flip side, this excellent agreement implies that mid-IR/THz photoexcitation creates a unique nonequilibrium state of hot, yet mostly conserved, Dirac fermions and bulk carriers in their respective bands. Third, the equilibrium surface and bulk scattering rates, *γ*_SS_ and *γ*_BS_ extracted before the photoexcitation, are 0.84 THz and 3.18 THz, respectively, which reveal an enhancement ratio, *γ*_BS_/*γ*_SS_ ~3.8. Remarkably, this value matches very well with the topological enhancement factor, 4, for protected, helical spin transport of non-interacting fermions in the presence of short-range disorder such as structural defects and surface or interface roughness. In the presence of long range, Coulomb disorder, this enhancement decreases from 4 to 2 due to increase of screening^31^. Furthermore, as shown in Fig. 2e, f, the scattering rates of the surface Dirac and bulk fermions nearly coincide with the frequency-dependent decay of Δ*σ*_1_(*ω*) for 2 meV and 7 meV probe, respectively. This corroborates again that tuning the THz probe frequency under mid-IR/THz pump can be used to disentangle the distinct symmetry-protected transport from the bulk conduction and measure their dynamics without reference to theoretical models. In between 2 and 7 meV, it consists of competing responses from surface and bulk, e.g., Δ*σ*_1_(*ω*) at 3 meV consists of THz conductivity contributions from both states with a similar strength.

### Wavelength-selective pumping from THz to visible

Figure 5 highlights ultra-broadband, wavelength-selective pumping that extends from THz and mid-IR to near-IR and visible. This data show that our THz conductivities following 4 meV (Fig. 5a) and 248 meV (Fig. 5b) pump have similar bipolar lineshape. In contrast, high-pump photon energy, e.g., 950 meV (Fig. 5d) and 1550 meV (Fig. 5e), show an overall positive conductivity change down to 2 meV. The clear similarities between mid-IR and THz pumping and their distinct difference vs. high-energy pumping allow us to distinguish two excitation channels, i.e., intraband (interband) excitations with low (high) photon energy pumping. Importantly, the data obtained for THz and mid-IR pumping can be well-fitted without changing carrier densities. In sharp contrast, the data for high-photon-energy pumping cannot be fitted in this way. Specifically, our good fittings obtained in Fig. 5a, b indicate that the pump-induced bulk components (dashed blue lines) have nearly identical amplitude and lineshape. This implies that the bulk responses following 4 meV and 248 meV pump are very similar, which indicate a possible transition from filled surface to empty bulk states following 248 meV pump cannot be a major channel, or at least any such contribution is much smaller in our probed THz spectral region than the main intraband signals. Additionally, it is fully consistent that the negative surface response (dashed green lines) is greater following 4 meV pump than 248 meV and 354 meV pump (still below but closer to the bulk bandgap) in the linear Dirac dispersion. In strong contrast, for high-photon-energy pumping we can see that the THz conductivities are positive in the probe range and much larger. Importantly, they cannot be fitted by the model with conserved carrier density any more, see Fig. 5d, e. Instead, they can be fitted decently only with Δ*n*_SS_ > 0 and Δ*n*_BS_ > 0, as shown in Fig. 5f, g. Their bulk (dashed blue lines) and surface (dashed green lines) components are both larger than their counterparts following low energy pump, which gives the overall positive THz conductivity change in the probed range, indicative of the bulk-dominant response. Moreover, please note that the fittings are faithful, because it only needs to change two scattering rates, which leads to distinctly different THz lineshapes that uniquely converge. We also rule out the possibility that two different sets of scattering rates, which differ from each other by >2%, can fit the same data. These results clearly demonstrate the control of THz conductivity by ultra-broadband, wavelength-selective pumping from THz to visible.

## Discussion

**Fig. 6:**
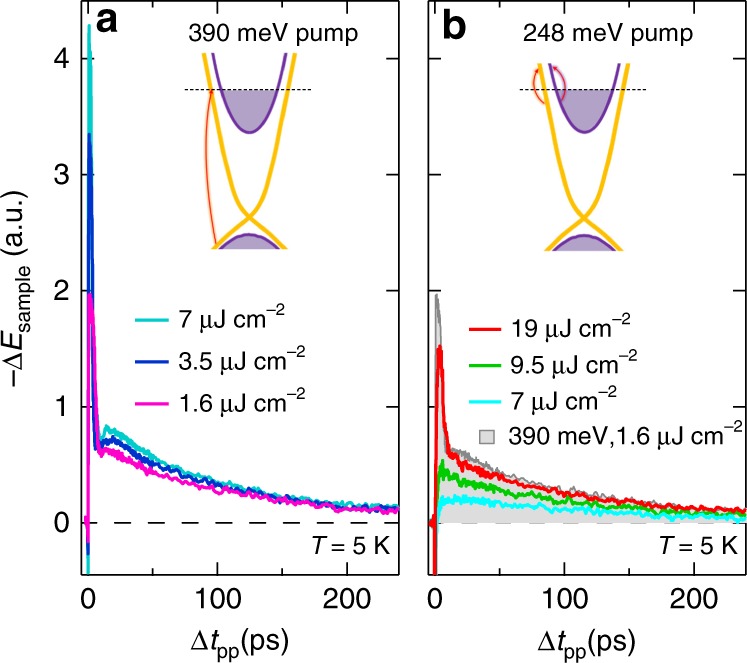
Ultrafast THz dynamics under interband and intraband photoexcitations. Pump-induced THz field changes Δ*E*_sample_ as a function of pump-probe delay Δ*t*_pp_ after **a** 390 meV (3.18 μm) and **b** 248 meV (5 μm) photoexcitations at various fluences and *T* = 5 K. The gate time *t*_gate_ is fixed at the peak of the static THz field shown in Fig. 1d. One of the THz traces from **a** (pink curve, 1.6 μJ cm^−2^) is also plotted in **b** as a filled gray curve for better comparison

To highlight the difference between the intraband excitation scheme used in this work and the interband excitation found in previous literature, Fig. 6a, b compare the ultrafast THz dynamics under 390 meV (3.18 μm) and 248 meV (5 μm) pumping, respectively. The former resonantly excites interband transitions in our bulk Bi_2_Se_3_ sample (Fig. 1a). Here, for simplicity, we show the THz field change Δ*E*_sample_ by fixing the gate time *t*_gate_ at the peak of the static THz field as a function of pump-probe delay Δ*t*_pp_, similar to previous studies^28,30^. Unlike the above-obtained frequency-dependent conductivity Δ*σ*_1_(*ω*) dynamics that closely follows the pump-induced change of surface/bulk scattering rates, Δ*E*_sample_ measured at a fixed gate time originates from frequency-integrated responses within the measured spectral range ~2–10 meV. This frequency average leads to more complicated dynamics that may come from all relaxation channels of opposite signs as shown in Fig. 2. Nevertheless, Fig. 6 shows that there is still a distinct difference between the interband (Fig. 6a) and intraband (Fig. 6b) pump excitation schemes. On the one hand, with resonant interband pump excitation at 390 meV (inset, Fig. 6a), Δ*E*_sample_ at several pump fluences shows two main decay features: (1) a dominant, fast overshoot, which exhibits fluence-dependent amplitude and quickly diminishes within ~4 ps; (2) a slow, hundreds of ps component, which is fluence independent, i.e., the Δ*E*_sample_ for all fluences merge and decay together after ~100 ps. On the other hand, with intraband excitation at 248 meV (inset, Fig. 6b), the fast overshoot component is absent in the THz responses at low fluences ≤12 μJ cm^−2^, the fluence used for mid-IR pumping in Figs. 1–4. A distinct difference is clearly visible between interband and intraband pumping with low fluence, e.g., the 7 μJ cm^−2^ (cyan) and 9.5 μJ cm^−2^ (green) traces. Interestingly, increasing the mid-IR pump fluence to 19 μJ cm^−2^ (red line in Fig. 6b) leads to a transition to a decay profile, where a fast overshoot component now appears similar to the interband photoexcitation, e.g., the 390 meV excitation scan at 1.6 μJ cm^−2^ (pink line in Fig. 6a). For a better comparison, this pink curve is also plotted in Fig. 6b as a filled gray curve, which resembles the 248 meV excitation scan at 19 μJ cm^−2^, unlike for the lower fluences. This reveals the same relaxation mechanism between the two, which can be attributed to the two photon absorption (TPA) present in the high fluence, mid-IR pumping case. This excites interband transitions in our sample in spite of below gap pump photon energy. The dynamics of Bi_2_Se_3_ under high-photon-energy pumping have been extensively studied recently and the few ps relaxation component is mainly from bulk-surface charge transfer and/or decay that can give the overshoot feature after interband carrier injection^16^. The absence of such process in the mid-IR pumping below ~12 μJ cm^−2^ is consistent with the intraband excitation channel and its similarity with THz (few meV) pumping.

Although the optical transition from filled surface state to empty bulk state with 248 meV mid-IR pump is potentially allowed, our results show that the relative strength of this channel and its contribution to the THz conductivity are, however, still an open question. The similarity between THz (few meV) and mid-IR pump implies that this channel is relatively weak for THz and mid-IR pumping when compared to the intraband excitation channel, unlike in the case of optical pump, which warrants further studies. In addition, our fitting is faithful because it only needs to change two parameters, which are the two time-dependent scattering rates directly seen in distinctly different time-resolved THz lineshapes. Since the fitting result is already uniquely converging with the two fitting parameters, additional parameters only relax the constraint and add uncertainty, which is hard to be physically meaningful. The mid-IR/THz pump spectroscopy experiment like ours reveals ultrafast manipulation of surface and bulk THz conductivity via wavelength-selective pumping and underpin the difference of intraband vs. interband pumping, shown in Fig. 5, without reference to any model and/or assumption for fitting. The observation of frequency-dependent hot carrier cooling times is shown in Fig. 2a–d, which directly distinguish the faster surface channel from the slower bulk in the raw data. These ultrafast conductivity dynamics of surface states are absent in the prior static THz and time-averaged probes.

Finally, we note that the assignment of THz conductivity of these two decay channels is justified by the distinctly different THz spectrum lineshapes and dynamics of the surface and bulk states, respectively. First, only the TI surface state could give rise to a photoexcited negative THz conductivity in Fig. 2e and intraband decay of Dirac spectrum at the surface is much faster than the bulk. Second, the other channel, i.e., photoexcited positive THz conductivity that we have assigned to bulk in Fig. 2f, exhibits a decrease in the scattering rate. This is well explained by the reduced ionized impurity scattering which leads to a decrease in scattering rate and an increase in THz conductivity at low frequency after photoexcitation. Third, a decrease in Bi_2_Se_3_ thickness has been shown to induce a transition from a bulk-like, positive conductivity to a surface-like, negative THz conductivity^30^.

In conclusion, we have provided the measurement and insights into the intraband, mid-IR, and THz pump-induced quantum transport in TIs. We achieve this by suppressing all interband transitions that mask the processes of main interest. The conclusions from this advanced spectroscopy are the demonstration of another tuning knob for both controlling THz conductivity and isolating the surface contribution from the bulk contribution, by using low photon energy pumping and comparing them to optical pumping. This scheme enables a clear observation of enhanced surface transport, which provides evidence for helical spin structure of surface (Dirac) electrons that suppresses backscattering. The pump photon energy tuning achieved in this way also allows us to control the surface transport optically in a selective way. Our experiments may evolve into a benchmark characterization method for high frequency topological transport in TI-based device development, and motivate fundamental quantum phase discovery and control at the boundaries of topology, magnetism^34^, and other broken symmetry states^35–37^.

## Methods

### Experimental schemes

Our mid-IR/THz pump and THz probe spectrometer is driven by a 1 kHz Ti:sapphire regenerative amplifier, which has 800 nm central wavelength and 40 fs pulse duration^38^. The majority of the output is used to pump either an optical parametric amplifier to generate mid-IR pulses tunable from 3–15 μm (or 83–413 meV) or a lithium niobate crystal to generate strong THz pulses centered at 4 meV^39^ allowing for selective intraband and interband photoexcitation. The other part of the output is used to generate and detect phase-locked THz electric fields in time-domain via optical rectification and electro-optic sampling in a 1 mm thick ZnTe crystal, respectively. THz fields with a bandwidth from 0.5 to 2.5 THz (2.1–10.3 meV) are used as a probe beam. The measurement scheme is briefly illustrated in Fig. 1a, in which mid-IR (red) or THz (yellow) pump photoexcites the sample and THz pulse (light blue) probes photoinduced responses of the sample. The transmitted THz probe containing spectral amplitude and phase information of the sample is directly measured in time-domain using an optical gate pulse. The setup is enclosed in a N_2_ gas purge box.

### Sample preparation and characterization

The Bi_2_Se_3_ thin film sample, 50 nm thick, is grown by molecular beam epitaxy on a 0.5 mm thick sapphire substrate. The sample is mounted together with a 0.5 mm thick pure sapphire substrate, used as a reference, into a cryostat with temperatures down to *T* = 5 K. Two copper mounts with the same aperture are placed directly in front of the sample and reference to ensure uniform photoexcitation and accurate comparison of their THz transmission.

The sample studied has a Fermi energy *E*_F_ ~ 60 meV from the bulk conduction band edge (Fig. 1a), as estimated from the measured plasma frequency in the static THz conductivity spectra shown in Fig. 1b. The gray lines are theoretical fittings that are discussed in Results. The estimated Fermi energy is consistent with the difference between the separately measured, interband optical transition onset *E*_inter_ ~ 390 meV using our wavelength-dependent pump-induced THz field change data (see Supplementary Note 1) and the bulk insulating bandgap *E*_g_ ~ 332 meV, which yields a similar *E*_F_ = *E*_inter_ − *E*_g_ ~ 58 meV. Please note that the exact value of the gap size is not critical for our scheme, nor does it change our conclusion, as long as the interband transition gap in our sample is larger than our pump photon energy, e.g., 4 meV and 248 meV used.

### THz data analysis

Raw THz field transients are measured as a function of gate time *t*_gate_ after transmission through (i) the reference bare substrate *E*_ref_(*t*_gate_), (ii) the unexcited sample *E*_sample_(*t*_gate_), and (iii) as pump-induced change Δ*E*_sample_(*t*_gate_, Δ*t*_pp_) at pump-probe delay Δ*t*_pp_. Figure 1c,d shows the 2D false-color plots of Δ*E*_sample_(*t*_gate_, Δ*t*_pp_) after below gap photoexcitation at 248 meV (5 μm) for high (300 K) and low (5 K) temperatures, respectively. Also plotted in the above figures are the corresponding *E*_sample_(*t*_gate_) (green curves) and pump-induced change Δ*E*_sample_(*t*_gate_) at Δ*t*_pp_ = 1 ps (blue curves). For the unexcited sample, through the fast Fourier transformation and Fresnel equation, the static THz conductivity, shown in Fig. 1b, is directly obtained from the experimentally measured complex transmission coefficient $\tilde t(\omega )$ with spectral amplitude and phase information extracted by comparing the fields transmitted through sample and reference, i.e., $\tilde t(\omega ) = E_{{\mathrm{sample}}}(\omega )/E_{{\mathrm{ref}}}(\omega )$, similar to the method reported in ref. ^29^ (see also Supplementary Note 5). For the excited sample at Δ*t*_pp_, the complex transmission coefficient is obtained by $\tilde t_{{\mathrm{excited}}}(\omega ) = [{\mathrm{\Delta }}E_{{\mathrm{sample}}}(\omega ) + E_{{\mathrm{sample}}}(\omega )]/E_{{\mathrm{ref}}}(\omega )$ and is used to extract the photoexcited transient THz conductivity. Afterwards, the pump-induced conductivity change ${\mathrm{\Delta }}\tilde \sigma (\omega ) = \tilde \sigma _{{\mathrm{excited}}}(\omega ) - \tilde \sigma (\omega )$ is calculated. The corresponding dielectric function can be calculated by the equation $\tilde \sigma (\omega ) = i[1 - \tilde \varepsilon (\omega )]\omega \varepsilon _0$. The simultaneously obtained real parts of transient conductivity change Δ*σ*_1_(*ω*) and dielectric function change Δ*ε*_1_(*ω*) as a function of Δ*t*_pp_ are presented in the study, which allow us to quantitatively describe the ultrafast dynamic evolution of the photoexcited Dirac and bulk fermions and their intrinsic scattering processes.

## Supplementary information

Supplementary Information

## Data Availability

The data that support the findings of this study are available from the corresponding author upon reasonable request.

## References

[1] Hsieh D (2008). A topological Dirac insulator in a quantum spin Hall phase. Nature.

[2] Zhang H (2009). Topological insulators in Bi_2_Se_3_, Bi_2_Te_3_ and Sb_2_Te_3_ with a single Dirac cone on the surface. Nat. Phys..

[3] Moore JE (2010). The birth of topological insulators. Nature.

[4] Hasan MZ, Kane CL (2010). Colloquium: topological insulators. Rev. Mod. Phys..

[5] Qi XL, Zhang SC (2010). The quantum spin Hall effect and topological insulators. Phys. Today.

[6] Koirala N (2015). Record surface state mobility and quantum hall effect in topological insulator thin films via interface engineering. Nano. Lett..

[7] Bowlan P (2017). Probing and controlling terahertz-driven structural dynamics with surface sensitivity. Optica.

[8] Hsieh D (2009). A tunable topological insulator in the spin helical Dirac transport regime. Nature.

[9] Wu C, Bernevig BA, Zhang SC (2006). Helical liquid and the edge of quantum spin hall systems. Phys. Rev. Lett..

[10] Kane CL, Mele EJ (2005). Z_2_ topological order and the quantum. Phys. Rev. Lett..

[11] Moore JE, Balents L (2007). Topological invariants of time-reversal-invariant band structures. Phys. Rev. B.

[12] Zyuzin AA, Burkov AA (2012). Topological response in Weyl semimetals and the chiral anomaly. Phys. Rev. B.

[13] Bravyi S, Hastings MB, Verstraete F (2006). Lieb-Robinson bounds and the generation of correlations and topological quantum order. Phys. Rev. Lett..

[14] Qu DX, Hor YS, Xiong J, Cava RJ, Ong NP (2010). Quantum oscillations and hall anomaly of surface states in the topological insulator Bi_2_Te_3_. Science.

[15] Wang YH (2012). Measurement of intrinsic dirac fermion cooling on the surface of the topological insulator Bi_2_Te_3_ using time-resolved and angle-resolved photoemission spectroscopy. Phys. Rev. Lett..

[16] Sobota JA (2012). Ultrafast optical excitation of a persistent surface-state population in the topological insulator Bi_2_Se_3_. Phys. Rev. Lett..

[17] Giorgianni F (2016). Strong nonlinear terahertz response induced by Dirac surface states in Bi_2_Se_3_ topological insulator. Nat. Commun..

[18] Kuroda K, Reimann J, Güdde J, Höfer U (2016). Generation of transient photocurrents in the topological surface state of Sb_2_Te_3_ by direct optical excitation with midinfrared pulses. Phys. Rev. Lett..

[19] Iyer V, Chen YP, Xu X (2018). Ultrafast surface state spin-carrier dynamics in the topological insulator Bi_2_Te_2_Se. Phys. Rev. Lett..

[20] Valdés Aguilar R (2012). Terahertz response and colossal Kerr rotation from the surface states of the topological insulator Bi_2_Se_3_. Phys. Rev. Lett..

[21] Wu L (2016). Quantized Faraday and Kerr rotation and axion electrodynamics of a 3D topological insulator. Science.

[22] Zhang Y (2010). Crossover of the three-dimensional topological insulator Bi_2_Te_3_ to the two-dimensional limit. Nat. Phys..

[23] Linder J, Yokoyama T, Sudbø A (2009). Anomalous finite size effects on surface states in the topological insulator Bi_2_Te_3_. Phys. Rev. B.

[24] Liu CX (2010). Oscillatory crossover from two-dimensional to three-dimensional topological insulators. Phys. Rev. B.

[25] Lu HZ, Shan WY, Yao W, Niu Q, Shen SQ (2010). Massive Dirac fermions and spin physics in an ultrathin film of topological insulator. Phys. Rev. B.

[26] Okada KN (2016). Terahertz spectroscopy on Faraday and Kerr rotations in a quantum anomalous Hall state. Nat. Commun..

[27] Dziom V (2017). Observation of the universal magnetoelectric effect in a 3D topological insulator. Nat. Commun..

[28] Valdés Aguilar R (2015). Time-resolved terahertz dynamics in thin films of the topological insulator Bi_2_Se_3_. Appl. Phys. Lett..

[29] Wu L (2013). A sudden collapse in the transport lifetime across the topological phase transition in (Bi_1−*x*_In_*x*_)_2_Se_3_. Nat. Phys..

[30] Sim S (2014). Ultrafast terahertz dynamics of hot Dirac-electron surface scattering in the topological insulator Bi_2_Se_3_. Phys. Rev. B.

[31] Ozturk T (2017). Influence of helical spin structure on the magnetoresistance of an ideal topological insulator. J. Phys. Commun..

[32] Yan B, Felser C (2017). Topological materials: Weyl semimetals. Annu. Rev. Condens. Matter Phys..

[33] Chattopadhyay D, Queisser HJ (1981). Electron scattering by ionized impurities in semiconductors. Rev. Mod. Phys..

[34] Wang J (2004). Ultrafast softening in InMnAs. Phys. E.

[35] Yang X (2018). Non-equilibrium pair breaking in Ba(Fe_1−*x*_Co_*x*_)_2_As_2_ superconductors: evidence for formation of photo-induced excitonic spin-density-wave state. Phys. Rev. Lett..

[36] Patz A (2017). Critical speeding up of nonequilibrium electronic relaxation near nematic phase transition in unstrained Ba(Fe_1−*x*_Co_*x*_)_2_As_2_. Phys. Rev. B.

[37] Patz A (2014). Ultrafast observation of critical nematic fluctuations and giant magnetoelastic coupling in iron pnictides. Nat. Commun..

[38] Luo L, Chatzakis I, Patz A, Wang J (2015). Ultrafast terahertz probes of interacting dark excitons in chirality-specific semiconducting single-walled carbon nanotubes. Phys. Rev. Lett..

[39] Yang X (2018). Terahertz-light quantum tuning of a metastable emergent phase hidden by superconductivity. Nat. Mater..

