# Supplementary material for: Ultrafast manipulation of topologically enhanced surface transport driven by mid-infrared and terahertz pulses in Bi2Se3
Source: Nat Commun. 2019 Feb 5;10:607. doi: 10.1038/s41467-019-08559-6 (PMC6363774; doi:10.1038/s41467-019-08559-6)
Supplement: Supplementary file 1 — Supplementary Information [file 41467_2019_8559_MOESM1_ESM.pdf]

Supplementary Information for
“Ultrafast Manipulation of Topologically Enhanced Surface Transport Driven by Mid-Infrared and Terahertz Pulses in Bi_2Se_3”

L. Luo[1], X. Yang[1], X. Liu[2], Z. Liu[1], C. Vaswani[1], D. Cheng[1], M. Mootz[3], X. Zhao[1], Y. Yao[1], C.-Z. Wang[1], K.-M. Ho[1], I. E. Perakis[3], M. Dobrowolska[2], J. K. Furdyna[2], and J. Wang[1*]

[1]*Department of Physics and Astronomy and Ames Laboratory-U.S. DOE, Iowa State University, Ames, Iowa 50011, USA.*

[2]*Department of Physics, University of Notre Dame, Notre Dame, IN 46556, USA.*

[3]*Department of Physics, University of Alabama at Birmingham, Birmingham, AL 35294-1170, USA.*

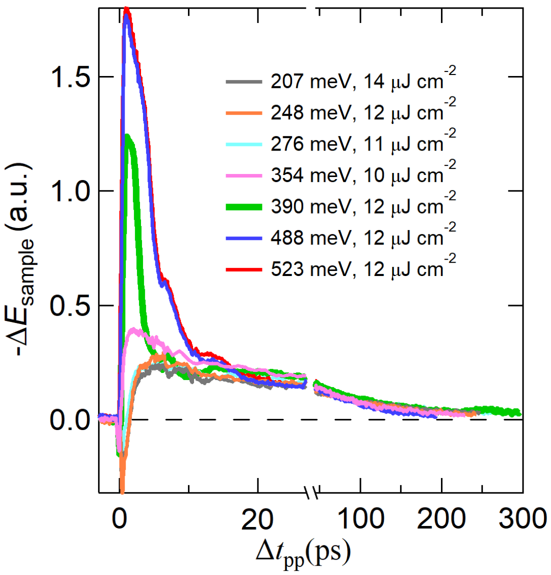

Supplementary Figure 1. **Pump wavelength dependent dynamics.** Ultrafast pump-induced THz field changes $\Delta E_{\mathrm{sample}}$ as a function of pump probe delay Δt_{pp} for several pump photon energies from 207 to 523 meV with similar pump fluence. The curves are scaled to match the amplitude at long decay time in order to compare their ultrafast dynamics at early times.

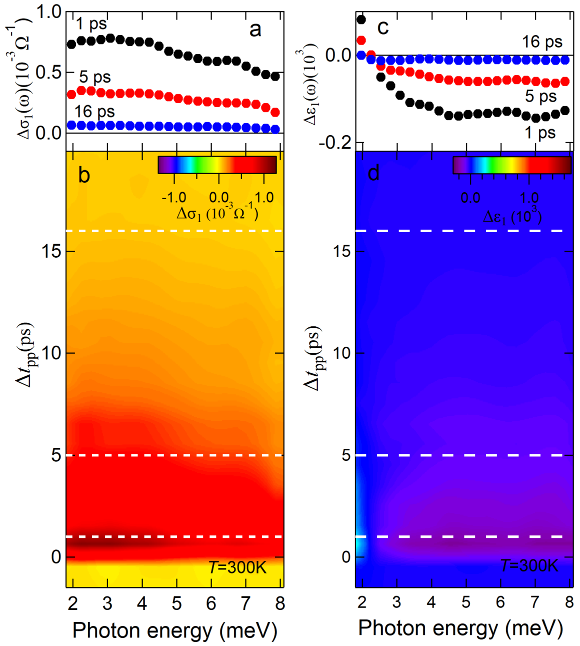

Supplementary Figure 2. **Pump-induced THz spectra at 300 K**. Pump-induced (**a**,**b**) $\Delta\sigma_1(\omega)$ and (**c**,**d**) $\Delta\varepsilon_1(\omega)$ after 248 meV (5 μm) photoexcitation with a fluence of 12 μJ cm^{-2} as a function of pump-probe delay $\Delta t_{\rm pp}$. (a) and (c) show the THz spectra from three cut positions from the corresponding 2D false-color plot of (b) and (d), respectively, at $\Delta t_{\rm pp}$=1, 5, 16 ps, as indicated by the horizontal white dashed lines.

Supplementary Table 1. Fitting parameters for low temperature THz spectra at 5 K following 248 meV pumping. Detailed fitting for Δt_{pp}=1, 9.4, and 15.4 ps are presented in Fig. 4 of the main text. The scattering rates are presented in Figs. 2e-2f of the main text.

	Drude 1 (static/pump off)		Drude 1 (excited/pump on)		Drude 2 (static/pump off)		Drude 2 (excited/pump on)	
Time delay(ps)	$(\omega_p)_1$ (THz)	γ_1(THz)	$(\omega_p)_1$ (THz)	γ_1(THz)	$(\omega_p)_2$ (THz)	γ_2(THz)	$(\omega_p)_2$ (THz)	γ_2(THz)
0.6	495	0.837	495	1.21554	330	3.18	330	2.92176
0.8	495	0.837	495	1.27389	330	3.18	330	2.86395
1.0	495	0.837	495	1.30521	330	3.18	330	2.81833
1.2	495	0.837	495	1.27389	330	3.18	330	2.81833
1.4	495	0.837	495	1.25382	330	3.18	330	2.80344
1.6	495	0.837	495	1.21554	330	3.18	330	2.81335
1.8	495	0.837	495	1.1623	330	3.18	330	2.86395
2.0	495	0.837	495	1.12933	330	3.18	330	2.85369
2.2	495	0.837	495	1.09066	330	3.18	330	2.86911
2.4	495	0.837	495	1.07592	330	3.18	330	2.8847
2.9	495	0.837	495	1.034	330	3.18	330	2.89519
3.4	495	0.837	495	1.00148	330	3.18	330	2.90576
4.4	495	0.837	495	0.959251	330	3.18	330	2.94336
5.4	495	0.837	495	0.925789	330	3.18	330	2.97081
6.4	495	0.837	495	0.915148	330	3.18	330	2.98754
7.4	495	0.837	495	0.912525	330	3.18	330	3.02155
8.4	495	0.837	495	0.884643	330	3.18	330	3.02442
9.4	495	0.837	495	0.874921	330	3.18	330	3.03885
10.4	495	0.837	495	0.867769	330	3.18	330	3.05342
11.4	495	0.837	495	0.860733	330	3.18	330	3.06222
12.4	495	0.837	495	0.855186	330	3.18	330	3.06812
15.4	495	0.837	495	0.847449	330	3.18	330	3.06812
18.4	495	0.837	495	0.840737	330	3.18	330	3.07999
21.4	495	0.837	495	0.829785	330	3.18	330	3.10401
24.4	495	0.837	495	0.827628	330	3.18	330	3.10401
29.4	495	0.837	495	0.825055	330	3.18	330	3.11616

Supplementary Note 1: More experimental data

Here we show more experimental results on pump-wavelength-dependent THz dynamics. Supplementary Fig. 1 shows ultrafast pump-induced THz field changes ΔE_{sample} as a function of pump probe delay Δt_{pp} for several pump photon energies from 207 to 523 meV with similar pump fluence so that the distinct ultrafast dynamics after intraband/interband photoexcitations can be identified fairly well. It shows clearly that when the pump photon energy is below 354 meV, ΔE_{sample} exhibits very similar dynamics without the dominant fast "overshoot" at early times which only appears for pump photon energy at 390 meV and above. The ultrafast dynamics changes drastically at 390 meV with the appearance of the fast "overshoot". Note that as discussed in Fig. 6 of the main text, the pump photon energy required for interband transition also highly depends on the fluence, so here we choose a fluence very similar to that we used throughout our study (248 meV, 12 μJ cm^{-2} as shown in Figs. 1-4) for comparison. It, together with Fig. 5 of the main text, clearly indicates that the 248 meV, 12 μJ cm^{-2} pump we used leads to below-gap intraband excitation.

In Supplementary Fig. 2, we plot pump-induced THz conductivity and dielectric function at T=300 K.

Supplementary Note 2: The Conservation of Carrier Density

We observe in the main text that the carrier concentrations of surface and bulk states are respectively conserved following the 248 meV mid-IR pump, implying that there is no significant interband transition from surface-to-surface or bulk-to-bulk states and we mostly drive surface-to-surface and bulk-to-bulk intraband transitions by using the 248 meV pump. There may be concerns that although 248 meV pump photon energy is well below the surface-to-surface and bulk-to-bulk interband transition gap, but it is still large enough to drive surface-to-bulk interband transition. This is an important issue and we would like to address it here.

Briefly, we believe that the optical transition from filled surface state to empty bulk state with 248 meV mid-IR pump in our case is potentially allowed. The relative strength of this channel and its contribution to the THz conductivity are, however, still an open question. Based on our

comprehensive pump wavelength dependent measurement results ranging from THz and mid-IR to near-IR and visible, we believe this channel is relatively weak.

First of all, it is clear that THz pump with 4 meV photon energy will mostly excite Fermi seas of surface and bulk states respectively, instead of driving any interband transitions. Our data in Fig. 5 shows that THz conductivities following THz (4 meV) and mid-IR (248 meV) pump have similar bipolar lineshape. In contrast, high pump photon energy, e.g., 950 meV and 1550 meV, shows an overall positive conductivity change down to 2 meV. The clear similarities between mid-IR and THz pumping and their distinct difference vs. high-energy pumping allow us to distinguish two excitation channels, i.e., intraband (interband) excitations with low (high) photon energy pumping, from raw conductivity data without reference to any model.

Second, our fitting shows that data obtained for THz and mid-IR pumping can be well-fitted without changing carrier densities. Specifically, our good fittings obtained in Figs. 5a-5b indicate that the pump-induced bulk components (dashed blue lines) have nearly identical amplitude and lineshape. This coincidence implies that the bulk responses following 4 meV and 248 meV pump are very similar, implying that these two contributions do share similar transition channels and strength for both THz and mid-IR pump. Therefore, a possible transition from filled SS to empty BS states following 248 meV pump, which is suppressed for 4 meV pump, cannot be a major channel, or at least any such contribution is much smaller in our probed THz spectral region than the main intraband signals. Additionally, it is fully consistent that the negative surface response (dashed green lines) is greater following 4 meV pump than 248 meV and 354 meV pump (still below but closer to the bulk bandgap) in the linear Dirac dispersion. On the other hand, for high photon energy pumping we can see that the THz conductivities are positive in the probe range and much larger. More importantly, they cannot be fitted by the model with conserved carrier density any more, see Figs. 5d-5e. Instead, they can be fitted well only with $\Delta n_{\rm SS} > 0$ and $\Delta n_{\rm BS} > 0$, see Figs. 5f-5g. Their bulk (dashed blue lines) and surface (dashed green lines) components are both larger than their counterparts following low energy pump. The clear similarities between mid-IR and THz pumping and their distinct difference vs. high energy pumping allow us to distinguish two excitation channels which we believe can only arise from the expected distinct contributions of intraband and interband excitation channels.

Last, we want to emphasize that our fitting is faithful, because it only needs to change 2 scat-

tering rates, which leads to distinctly different THz lineshapes: one of them (dashed green lines) has bipolar shape, which mostly determines the overall lineshape, while the other one (dashed blue lines) mostly determines the amplitude. Therefore, the fitting result is uniquely converging, and we rule out the possibility that two different sets of scattering rates, which differ from each other by >2%, can fit the same data.

Supplementary Note 3: The assignment of THz conductivity to surface and bulk bands

In our scheme, we directly measure a complete 2D data set of conductivity dynamics in the conjugated spectral-temporal domain in Figs. 2a-2d, and we provide the first observation of frequency-dependent decay dynamics of THz spectra at low temperature in Figs. 2e-2f that directly show the existence of both the surface and bulk channels in the time domain raw data, without references to any model or fitting. As we discuss in the main text, the experimentally obtained frequency-resolved dynamics in $\Delta\sigma_1$ probed at 2 meV can be used to directly measure surface transport dynamics, which is very different from bulk conduction at 7 meV. The assignment of THz conductivity of these two channels is further justified by the distinctly different THz spectrum lineshapes and dynamics of the surface and bulk states, respectively, as further discussed below.

One of the most relevant examples is shown in Ref. [1] which reports thickness-dependent THz conductivity of Bi_2Se_3. With 5 QL thin film, where the surface response is dominant, the THz conductivity is purely negative. With increasing thickness, the contribution from bulk becomes larger, and therefore, the THz conductivity changes to positive. The data clearly shows a transition from a bulk-like positive THz conductivity to a surface-like negative THz conductivity by decreasing the thickness of Bi_2Se_3. Our assignment based on our THz and mid-IR pumping data is consistent with this paper.

In addition, the Bi_2Se_3 surface scattering rate was found to be ∼1-1.5 THz in most literature [2]. The lower scattering rate we have assigned to TI surface state is 0.84 THz, very close to the reported range. While the higher scattering rate we measured (∼3.18 THz) is too large. Compared to the bulk state, the TI surface state should have a lower scattering rate due to the topological protection of surface carriers. Our assignment is consistent with this paper too.

Supplementary Note 4: The contribution of 2DEG to the THz conductivity

The 2DEG has been observed to coexist with the topological state on the surface of TIs due to a defect-induced surface band bending effect. So far, the 2DEG has been directly observed by the angle-resolved photoemission spectroscopy [3, 4]. However, no THz study of n-type Bi_2Se_3 samples has clearly reported to observe or attributed any part of THz conductivity to 2DEG yet [1, 2, 5]. Ref [5] assigned the THz conductivity to (1) topological surface state and (2) bulk or accumulation layer. Ref [1] assigned the THz conductivity to (1) the surface and (2) bulk states. Ref [2] also assigned it to the (1) surface and (2) bulk states. It also stated that 2DEG does not play a significant role in their THz measurements. In addition, another study [6] did not find 2DEG feature from THz responses for Bi_2Se_3 thin films ranging from 16-100 QL either and speculated that 2DEG states have mobilities so low that they do not make appreciable contributions to (THz) transport.

Given the above literature results, one can conclude that a common agreement in the THz community is that the 2DEG does not contribute significantly to the measured THz conductivity. We believe that there are several possible reasons for this: (1) the 2DEG density is much smaller than that of the TI surface and bulk states. (2) The 2DEG may only contribute an insignificant THz contribution in the measured spectral range of 0.5-2.5 THz (2.1-10.3 meV). Indeed, as shown in [7], the 2DEG density is only 1/4 of the TI surface carriers and the 2DEG mobility is 6 times larger than that of TI surface carriers for Bi_2Se_3 samples with a thickness of $\sim$8-256 QL. These will lead to a much smaller 2DEG spectral weight, which resides mostly below the lower limit of our THz probe range of 0.5 THz ($\sim$2.1 meV).

Supplementary Note 5: THz conductivity calculation and fitting parameters

The complex THz conductivity $\widetilde{\sigma}(\omega)$ is calculated from the measured transmission $\widetilde{t}(\omega) = E_{\text{sample}}(\omega)/E_{\text{ref}}(\omega)$ similar to the equation reported in Ref. [5], i.e., $\widetilde{t}(\omega) = (1+n)/(1+n+Z_0\widetilde{\sigma}(\omega)d)\exp^{i\phi}$, where ϕ is the measured phase difference between sample and reference, n is the substrate refractive index, d is the thickness of the sample, and Z_0=377 Ω.

The fitting details using Eq. 1 are presented in Fig. 4 of the main text. The extracted scattering rates of SS and BS are presented in Figs. 2e-2f. All fitting parameters are listed in Supplementary Table 1.

Supplementary References

[1] Sim, S. et al. Ultrafast terahertz dynamics of hot Dirac-electron surface scattering in the topological insulator Bi_2Se_3. *Phys. Rev. B* **89**, 165137 (2014).

[2] Valdes Aguilar, R. et al. Time-resolved terahertz dynamics in thin films of the topological insulator Bi_2Se_3. *Appl. Phys. Lett.* **106**, 011901 (2015).

[3] Bianchi, M. et al. Coexistence of the topological state and a two-dimensional electron gas on the surface of Bi_2Se_3. *Nat. Commun.* **1**, 128 (2010).

[4] King, P. D. C. et al. Large Tunable Rashba Spin Splitting of a Two-Dimensional Electron Gas in Bi_2Se_3. *Phys. Rev. Lett.* **107**, 096802 (2011).

[5] Wu, L. et al. A sudden collapse in the transport lifetime across the topological phase transition in $(Bi_{1-x}In_x)_2Se_3$. *Nat. Phys.* **9**, 410 (2013).

[6] Valdes Aguilar, R. et al. Terahertz Response and Colossal Kerr Rotation from the Surface States of the Topological Insulator Bi_2Se_3. *Phys. Rev. Lett.* **108**, 087403 (2012).

[7] Bansal, N., Kim, Y. S., Brahlek, M., Edrey, E. & Oh, S. Thickness-Independent Transport Channels in Topological Insulator Bi_2Se_3 Thin Films. *Phys. Rev. Lett.* **109**, 116804 (2012).
